# Supplementary material for: Analysis of the Impact of Environmental and Agronomic Variables on Agronomic Parameters in Soybean Cultivation Based on Long-Term Data
Source: Plants (Basel). 2022 Oct 30;11(21):2922. doi: 10.3390/plants11212922 (PMC9656608; doi:10.3390/plants11212922)
Supplement: Supplementary file 1 [file plants-11-02922-s001.zip › plants-1967574-supplementary.pdf]

Supplementary Information

Table S1. Selected soybean cultivars which were most frequently used in the experiments and their grain yield (the cultivars are ranked according grain yield, from the highest grain yield).

Name of cultivar	Number of experiments	Grain yield ($Mgha^{-1}$)
Acardia	83	3.928
Kofu	85	3.780
Obelix	83	3.603
Sirelie	99	3.595
ES Comandor	111	3.561
Moravians	92	3.477
Abelina	135	3.454
Viola	102	3.445
Petrina	113	3.402
Aligator	111	3.259
Au relin a	85	3.170
Mavka	89	3.092
Adessa	88	3.068
Erica	123	2.962
